# 3D Flower-Like NiO Hierarchical Structures Assembled With Size-Controllable 1D Blocking Units: Gas Sensing Performances Towards Acetylene

**DOI:** 10.3389/fchem.2018.00472

**Published:** 2018-10-11

**Authors:** He Zhang, Wei-Gen Chen, Yan-Qiong Li, Ling-Feng Jin, Fang Cui, Zi-Hao Song

**Affiliations:** ^1^State Key Laboratory of Power Transmission Equipment & System Security and New Technology, School of Electrical Engineering, Chongqing University, Chongqing, China; ^2^School of Electronic and Electrical Engineering, Chongqing University of Arts and Sciences, Chongqing, China

**Keywords:** NiO, hierarchical structures, blocking units, sensor, gas sensing performances

## Abstract

Acetylene gas (C_2_H_2_) is one of the main arc discharge characteristic gases dissolved in power transformer oil. It is of great potential to monitor the fault gas on-line by applying gas sensor technology. In this paper, gas sensors based on nanorods and nanoneedles assembled hierarchical NiO structures have been prepared. Herein, we focus on investigate the relationship between the sizes of the assembling blocking units and gas sensing properties. It can be found that the addition of CTAB/EG plays a vital role in controlling the sizes of blocking unit and assembly manner of 3D hierarchical structures. A comparison study reveals that an enhanced gas sensing performance toward C_2_H_2_ for the sensor based on nanoneedle-assembled NiO flowers occurs over that of nanorod-assembled NiO. This enhancement could be ascribed to the larger specific area of needle-flower, which provides more adsorption and desorption sites for chemical reaction as well as effective diffusion channels for C_2_H_2_. Besides, a method of calculating the specific surface area without BET testing was presented to verify the results of gas sensing measurement. The possible growth mechanism and gas sensing mechanism were discussed. Such a synthesis way may open up an avenue to tailor the morphologies and control the sizes of blocking units of some other metal oxides and enhance their gas sensing performances.

## Introduction

As we all know, the stable and reliable operation of power transformers is particularly important for the safety and stability of power system. When the oil-immersed power transformers work for a long time, the insulating oil, and paper will gradually deteriorate and produce various trace characteristic gases, which actually dissolve in transformer insulation oil (Singh and Bandyopadhyay, 2010). When the power transformer has the spark of oil or arc discharge fault, it will generate the fault characteristic gas with acetylene gas (C_2_H_2_) as the main component. It is of great potential to monitor the fault gas on-line by applying gas sensor technology. The monitoring of C_2_H_2_ gas content can predict the internal latent failure of the transformer (Chen et al., 2013; Jin et al., 2017). So, C_2_H_2_ gas is selected as the target gas in this paper.

A common method for detecting C_2_H_2_ gas dissolved in transformer oil is metal oxide semiconductor (MOS) based gas sensor (Zhu and Zeng, 2017). Among various MOS sensing materials, nickel oxide (NiO) as a wide band gap (Eg = 3.6–4.0 ev) p-type semiconductor has taken a dominated position due to its outstanding physical and chemical properties. Recently, NiO has been extensively applied in multifarious application fields such as electrode materials (Zhang et al., 2004), solar cells (Nakasa et al., 2005), catalysts (Kaminski et al., 2018), and gas sensors (Cao et al., 2015a; Yu et al., 2017).

It's believed that one-dimensional (1D) nanostructures with large surface to volume have great potential to improve the sensing properties. However, there are some shortcomings for 1D structure, i.e., inevitable serious stacking configuration and thermal/ chemical instability. Given this, the sensing materials can be designed into three–dimensional (3D) hierarchical structure assembled by 1D blocking units, which not only prevents the 1D blocking units from serious stacking but also inherits the merits of 1D nanomaterials (Duo et al., 2016; Zhu et al., 2018). The ability to control the assembly configuration, the morphology and size of building units in hierarchical architectures is of utmost importance for the realization of multifunctional nanodevices (Kim and Yong, 2011). Recently, assembly of 1D blocking units into hierarchical structures has been a hot topic in the research. But there are few explorations about the synthesis of hierarchical structures with size-controllable blocking units and studying the influence of the size of the assembling units on gas sensing performances.

In this paper, nanorods, and nanoneedles assembled 3D flower-like NiO hierarchical structures were successfully synthesized via hydrothermal synthesis. Herein, we focus on investigate the relationship between the sizes of the assembling blocking units and gas sensing properties. It can be found that the addition of CTAB/EG plays a vital role in controlling the sizes of blocking unit and assembly manner of 3D hierarchical structures. A comparison study reveals that an enhanced gas sensing performance toward C_2_H_2_ for the sensor based on nanoneedle-assembled NiO flowers occurs over that of nanorod-assembled NiO. In order to shed light on this phenomenon, a method of calculating the specific surface area without BET testing was presented to verify the results of gas sensing measurement. Based on our experimental results, the possible formation mechanism of two kinds of NiO nanoflowers is primarily discussed. It's expected that this study can promote the development of gas sensing materials via lower dimensional assembly.

## Experimental

### Synthesis of the nanorods-assembled hierarchical NiO nanoflowers

In a typical experiment of nanorods assembled NiO nanoflowers, 0.4 g of Ni(NO_3_)_2_·6H_2_O was added to 40 ml of distilled water under vigorous stirring for 10 min. 0.18 g of cetyltrimethyl ammonium bromide (CTAB) was introduced into the above solution. Then, under continuous magnetic stirring, ammonia (NH_3_·H_2_O, 25%) was dripped into the mixed solution to obtain the pH = 9. After thorough mixing, the resulting mixture was transferred to a 50 ml autoclave and maintained overnight at 180°C. After cooling naturally, the precipitates were washed sequentially and dried in air at 60°C. Finally, the powder was calcined at 500°C for 2 h. The sample was labeled as rod-flower.

### Synthesis of the nanoneedles-assembled hierarchical NiO nanoflowers

Typically, 0.4 g of NiCl_2_·6H_2_O and 0.08 g of Na_2_C_2_O_4_ were poured into 15 ml of distilled water. Then 25 ml ethylene glycol (EG) was added into the solution with sequentially stirring. The mixed solution was loaded into a 50 ml autoclave and heated to 160°C for 12 h. The subsequent process including centrifugation, washing, drying and calcining are the same as the above. The sample after annealing was designated as needle-flower.

### Characterization

Crystal structure of as-prepared samples was examined through X-ray diffraction (XRD, D/Max-1200X, Rigaku). The surface morphologies and nanostructures of the samples were inspected by scanning electronic microscopy (SEM, JEM-6700F) and transmission electron microscopy (TEM, JEM-1200EX).

The detailed process about the fabrication of planar gas sensor and gas-sensing test is as follows Jin et al. (2017). Firstly, the appropriate amount of as-prepared NiO powders was fully ground and mixed with diethanolamine and ethanol to form a slurry suspension. The pastes were evenly coated onto the electrodes of sensor's substrate. Then, the sensor was placed in aging platform and maintained at 120°C for 100 h to improve the stability of the sensor. Gas sensing properties toward C_2_H_2_ were measured using a CGS-1TP (Chemical Gas Sensor-1 Temperature Pressure) intelligent gas sensing analysis system. The sensor was placed on the heating table of the gas chamber and two probes were adjusted to ensure good electrical signals of the sensing materials. Then, the working temperature was set and air was delivered into the chamber at a constant flow rate. When the resistance of the sensor was stable in the air, it's denoted as Ra. Then, a certain amount of target gas was injected into the chamber through the injection hole. The change of resistance curve in the software was observed until the resistance value was stable again, denoted as Rg. The target gas flow was vented and the sensor was exposed to air again. The concentration of target gas (C_2_H_2_) was controlled by the mass flow controllers (MFC) with the following equation (Equation 1):

(1) $$\begin{array}{r} \text{Gas concentration }(\text{ppm})\\ =\frac{\text{Flow rate }(\text{target gas})\times \text{Gas cylinder }(\text{target gas})}{\text{Flow rate }(\text{target gas})+\text{Flow rate }(\text{air})} \end{array}$$

The response (S) of the sensor was defined as the ratio of Rg to Ra. And the response (recovery) time was regarded as the time required reach 90% of the total resistance change.

## Results and discussion

### Morphology and structure

**Figure 1:**
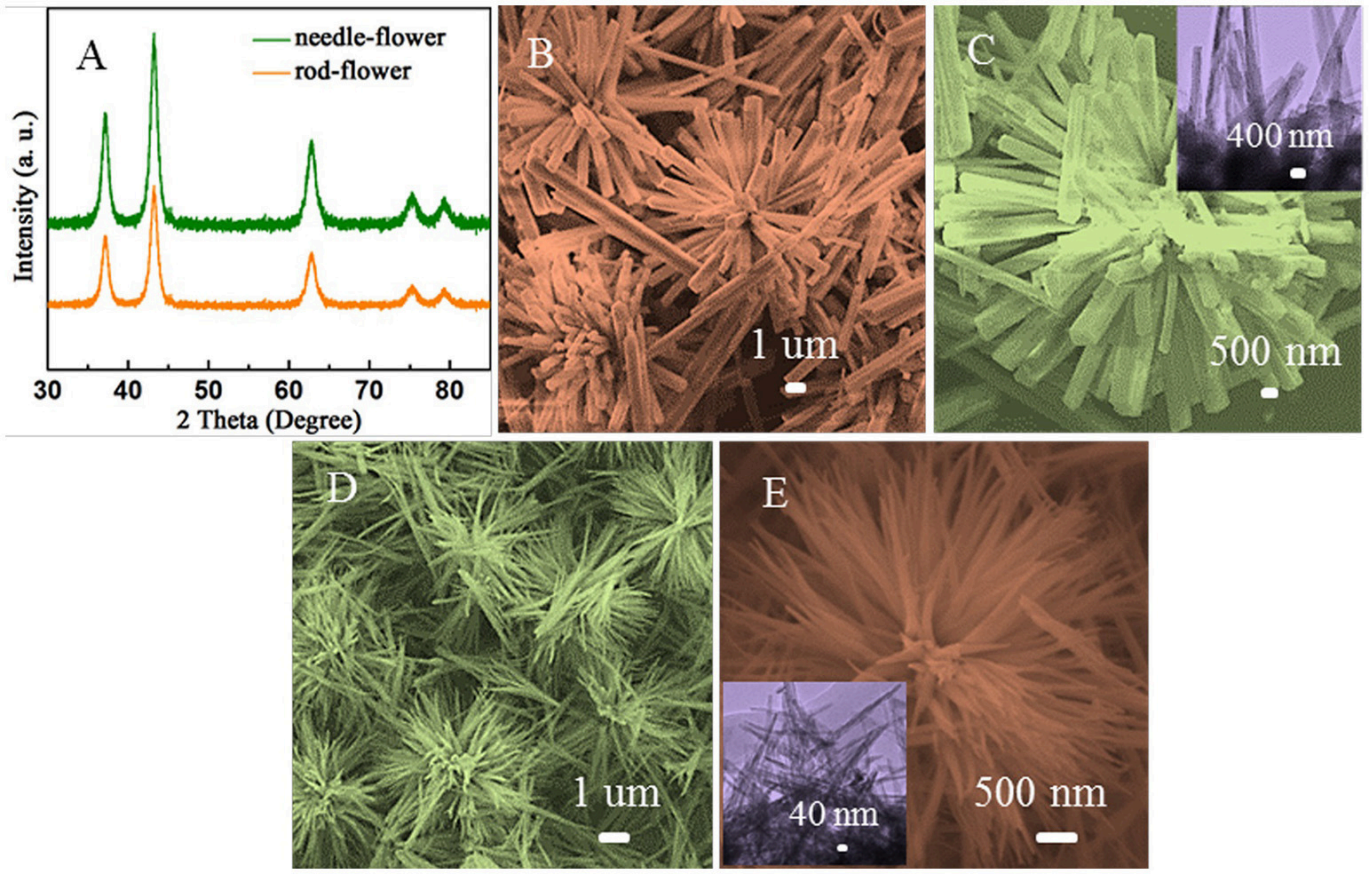
**(A)** XRD patterns of the obtained samples. **(B,C)** SEM images of the rod-flower NiO and TEM image in the inset of **(C)**. **(D,E)** SEM images of the needle-flower NiO and TEM image in the inset of **(E)**.

Figure 1A shows the XRD patterns of the obtained samples. The identified peaks in two curves can be well matched with the cubic crystalline structure of NiO (JCPDS Card no. 04-0835) without observable impurity peaks, demonstrating that high purity of NiO. Morphologies and structural features of the samples are characterized by SEM and TEM, as shown in Figures 1B–E. From Figure 1B, the NiO hierarchical nanoflowers are assembled from a bunch of well-defined nanorods. The roots of these nanorods come together while the tips are detached. Figure 1C illustrates that each individual nanorod from the flower shared the same geometric center. The average diameter of these nanorods is ~900 nm and the length is ~6 μm. Additionally, some rods are scattered around flower-like structures. As observed in the inset of Figure 1C, the size of nanorods was similar to what we had observed in SEM images. In Figure 1D, the nanoneedles are assembled into homogeneously distributed flower-like structures (AlHadeethi et al., 2017). The magnified SEM image in Figure 1E displays the nanoneedles are thicker at roots with sharper emanative ends. Each needle is ~2.5 um in length and ~80 nm in diameter at the middle, which is in consistence with the observation in TEM image (the inset of Figure 1E).

### Formation mechanism

**Figure 2:**
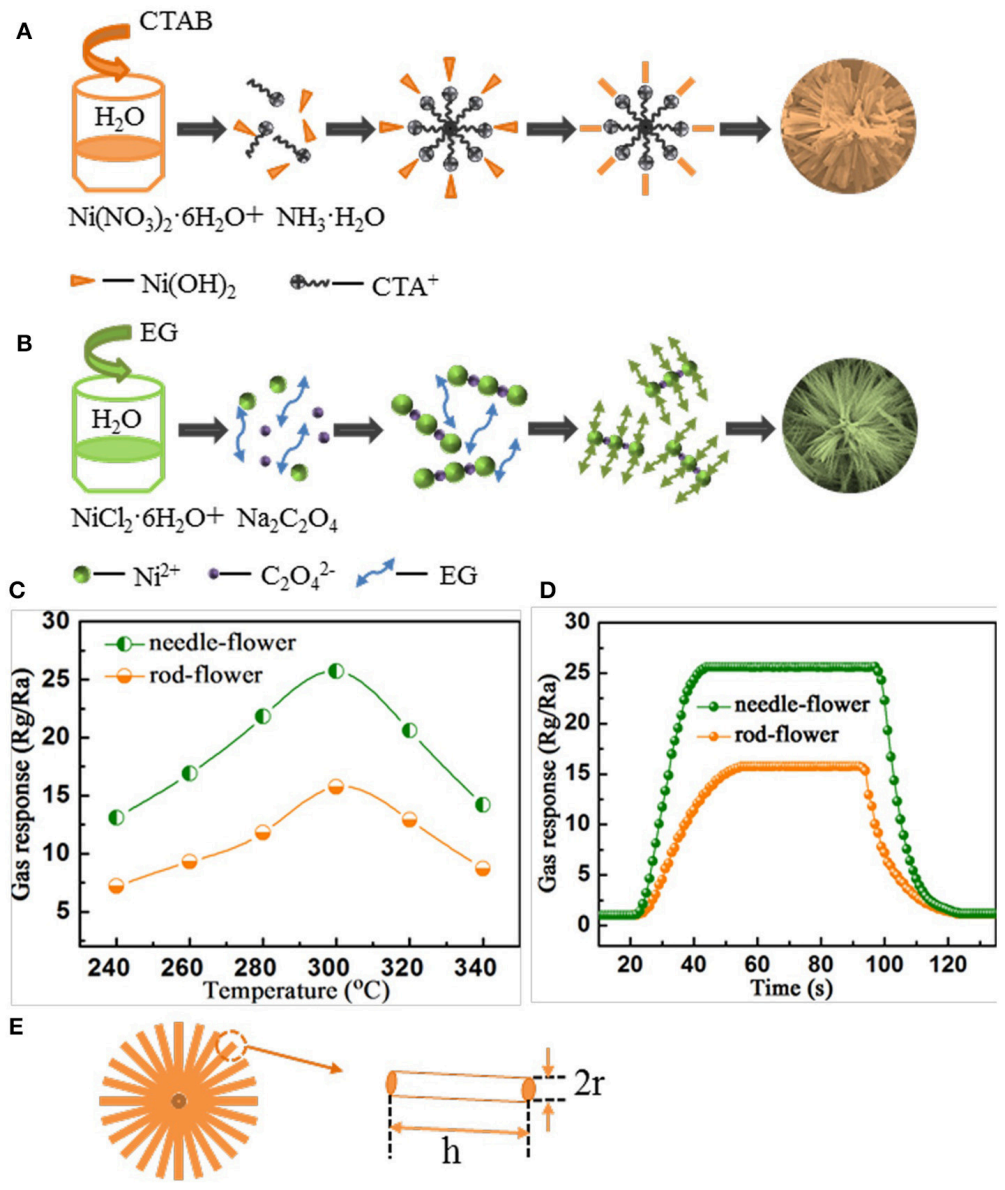
**(A,B)** Schematic of the formation process. **(C)** Gas response as the function of operating temperature under C_2_H_2_ concentration of 200 ppm. **(D)** The response and recovery characteristics of rod-flower and needle-flower NiO under C_2_H_2_ concentration of 200 ppm at 300°C. **(E)** A simplified model to calculate the surface area of hierarchical NiO nanoflowers.

Based on the above observations, we proposed a possible formation mechanism for the morphologies evolution of the nanorod-assembled NiO nanoflowers, as shown in Figure 2A. Firstly, ammonia aqueous acts as an alkaline reagent to release OH^−^ ions. CTAB is a surfactant with a hydrophobic part (Li Y. Q. et al., 2015; Liu et al., 2017). When the Ni(OH)_2_ comes across CTAB, Ni(OH)_2_ will be preferably absorbed on the CTA^+^ heads. Subsequently, the grown Ni(OH)_2_ nanoparticles are connected with each other by orientation attachment to form many nanorods. It's proposed that CTAB seemingly acts as an adhesive to gather the nanorods together (Li T. M. et al., 2015; Miao et al., 2017). Finally, the nanorods self-assemble into the ultimate flower-like architectures driven by the minimum surface energy theory.

For the formation of the nanoneedle-assembled hierarchical NiO nanoflowers (Figure 2B), firstly, Ni^2+^ and C_2_${\text{O}}_{4}^{2-}$ can be illustrated as a NiC_2_O_4_·2H_2_O polymer type ribbon owing to the complexation of Ni^+^ and C_2_${\text{O}}_{4}^{2-}$. EG is a surfactant with symmetrical structures and functional group-OH, which serves as a ligand to Ni and blocked the crystal surface paralleled to [0,1,1] direction (Cao et al., 2015b). And then the above microstructures are connected with each other along [0,1,1] direction fabricate the needle-like structures. With the reaction time goes by, NiC_2_O_4_·2H_2_O nanoneedles aggregate with each other to assemble into hierarchical needle-flower. Finally, NiO nanoflowers are obtained by thermal calcination.

### Gas sensing properties

To further study the connection between the size of the assembling units and gas sensing performances, we conduct gas sensing experiments. Firstly, we investigate gas response curves with temperature changing toward 200 ppm C_2_H_2_. In Figure 2C, there is a volcano-shaped trend for the changes in gas response of both kinds of nanoflowers. Apparently, the gas response of the needle-flower NiO is higher than that of rod-flower. The responses at peaks are 15.76 and 25.71 at 300°C, respectively (Long et al., 2018; Zhang et al., 2018). Herein, we determine the optimum working temperature to be 300°C for the later testing. Figure 2D demonstrates the response and recovery characteristic of hierarchical NiO nanoflowers toward 200 ppm C_2_H_2_ at 300°C. Both sensors exhibit excellent gas sensing performances. It's clearly seen that the response of the needle-flower NiO is higher than that of rod-flower. Besides, the needle-flower NiO exhibits a shorter response and recovery time (23 and 26 s) compared with that of the rod-flower NiO (34 and 32 s). In addition, a comparison about the sensing performances of NiO sensors in this work and other literature reports is summarized in Table 1. It is not difficult to find that the needle-flower NiO based gas sensor in this paper has excellent gas sensing characteristics and has great potential to be a promising candidate for gas-sensitive materials (Lin et al., 2015; Lu et al., 2016; Majhi et al., 2018; San et al., 2018).

**Table 1:** Comparison of NiO based gas sensor in this work and those literature reports.

**Sensing materials**	**Target gas (ppm)**	**Temperature (°C)**	**Response**	**References**
Needle-flower NiO	Acetylene 200 ppm	300	25.71	This work
Rod-flower NiO	Acetylene 200 ppm	300	15.76	This work
Hollow NiO/SnO_2_ heterostructure	Acetylene 100 ppm	206	13.8	Lin et al., 2015
Porous cactus-like NiO	Acetone 100 ppm	260	13.51	Lu et al., 2016
NiO/ZnO heterojunction microflowers	Formaldehyde 100 ppm	200	13.1	San et al., 2018
Pristine NiO nanoparticles	Ethanol 100 ppm	300	1.88	Majhi et al., 2018
Core-shell Au@NiO	Ethanol 100 ppm	200	2.54	Majhi et al., 2018

Whether the working temperature vs. response or the response and recovery characteristic, sensor based on needle-flower NiO absolutely prevails over that of rod-flower. This may be attributed to the needle-flower's high surface area. In order to verify this hypothesis, we use a simple simplified model to calculate the surface area of the hierarchical NiO structures (Figure 2E) from associated literature (Lee, 2009; Zhang et al., 2012). We made a slight change according to our data based on the theory. Whether constituent blocking units are nanorods or naononeedles, this proposal has reasonable guiding significance to conduct qualitative analysis. In this modified model, the specific surface area (Equation 2) is

(2) $$\begin{array}{r} \text{S}≅\frac{\left(\pi {\text{r}}^{2}+2\pi \text{rh}\right)\text{n}}{\text{n}\pi {\text{r}}^{2}\rho }\sim \frac{1}{\rho }\left(1+\frac{2\text{h}}{\text{r}}\right) \end{array}$$

Where S stands for the specific surface area, r is the equivalent radius of 1D unit, h is the length of 1D unit which can be also expressed as the radius of hierarchical structures, n is the number and ρ is the density of 1D unit. To a specific material, ρ can be considered as a constant. So S is proportional to h/r. Through the measurement and calculation, the h/r value (66.7) of the nanoneedles is ~5 times that of the nanorods (13.4). So the S of the needle-flower is larger. It can explain why the needle-flower NiO shows higher gas response and rapid response/recovery behavior. The larger specific area will provide many adsorption and desorption sites for oxygen, leading to the increasement in the conductivity.

### Gas sensing mechanism

The sensing mechanism of NiO-based gas sensors involves three serial reactions: adsorption-oxidation-desorption (Zhu et al., 2017). In the case of p-type semiconductor, its carrier is the hole with positive charge. Specifically, when the sensor is in the air, oxygen molecules react with NiO surface (Equations 3, 4). Due to the above reaction, electrons on the NiO surface combine with O_2_ to form oxygen negative ions (${\text{O}}_{2}^{-}$, O^−^, and O^2−^). This process cause the decrease of electrons and the increase of holes to form a hole accumulation layer, resulting in the resistance of the sensor decreases correspondingly. When NiO surface comes into contact with C_2_H_2_ gas, oxygen ions will oxidize gas molecules into CO_2_ and H_2_O, and releases electrons to recombine with holes (Equations 5, 6), leading to the decrease of carriers in hole accumulation layer and an increase in the resistance (Balamurugan et al., 2014; San et al., 2015).

(3) $$\begin{array}{rcl} {\text{O}}_{2}\;\left(\text{gas}\right) & \to & {\text{O}}_{2}\;\left(ads\right) \end{array}$$

(4) $$\begin{array}{rcl} {\text{O}}_{2}\;\left(\text{ads}\right)+\text{n}{\text{e}}^{-} & \to & {\text{O}}^{\text{n}-}\;\left(\text{ads}\right) \end{array}$$

(5) $$\begin{array}{rcl} {\text{C}}_{2}{\text{H}}_{2}\;\left(\text{gas}\right) & \to & {\text{C}}_{2}{\text{H}}_{2}\;\left(\text{ads}\right) \end{array}$$

(6) $$\begin{array}{rcl} {\text{C}}_{2}{\text{H}}_{2}\;\left(\text{ads}\right)+{\text{O}}^{\text{n}-}{\left(\text{ads}\right)}^{-} & \to & \text{C}{\text{O}}_{2}+{\text{H}}_{2}\text{O}+\text{n}{\text{e}}^{-} \end{array}$$

## Conclusion

In summary, nanorods and nanoneedles assembled NiO hierarchical structures have been successfully synthesized via a hydrothermal method and annealing process. Based on the comparative studies, we draw a conclusion that the size (length and diameter) of blocking units has a great influence on gas sensing properties of hierarchical structures. The integral morphologies and sizes of blocking units can be controlled by tuning the additives. Here, CTAB/EG was introduced as a structure-directing agent to regulate the aggregation and assembly. Compared with rod-flower NiO, the needle-flower NiO based sensor exhibits an enhanced gas sensing performance. This enhancement could be ascribed to the larger specific area of needle-flower, which provides more adsorption and desorption sites for chemical reaction as well as abundant effective diffusion channels for C_2_H_2_. The results hold a novel point in constructing highly efficient gas sensors. The detection capability of gas sensors determines the effectiveness of transformer on-line monitoring. Therefore, optimize the morphology and structure of gas sensitive materials is very meaningful work. Gas sensors with the advantages of miniaturization structure, high sensitivity, and fast response speed have very high practical value in power system security.

## Author contributions

HZ and W-GC conceived and designed the experiments. HZ, Y-QL, and L-FJ performed the experiments. FC and Z-HS analyzed the data. HZ wrote the manuscript with input from all authors. All authors read and approved the manuscript.

### Conflict of interest statement

The authors declare that the research was conducted in the absence of any commercial or financial relationships that could be construed as a potential conflict of interest.
